# Soft Tissue Augmentation in Periodontics and Implantology: Current Concepts, Techniques, and Clinical Outcomes

**DOI:** 10.7759/cureus.109501

**Published:** 2026-05-23

**Authors:** Soamya Gandhi, Mayur Kaushik, Shivi Khattri, Soundarya Singh

**Affiliations:** 1 Department of Periodontology, Subharti Dental College and Hospital, Swami Vivekanand Subharti University, Meerut, IND

**Keywords:** angiogenesis, connective tissue biology, dental implant(s), periodontal tissues/periodontium, scaffolds, tissue engineering

## Abstract

Oral soft tissue augmentation plays a crucial role in enhancing esthetics, managing gingival recession, establishing an adequate band of keratinized mucosa, and supporting both periodontal and implant therapy. Traditionally, autogenous grafts such as free gingival grafts (FGGs) and subepithelial connective tissue grafts (SCTG) have been considered the gold standard due to their predictable outcomes and long-term stability.

However, their limitations, including donor site morbidity, increased surgical time, and limited tissue availability, have driven the development of alternative biomaterials. In recent years, soft tissue substitutes (STSs), particularly scaffold-based matrices derived from human and animal sources, have gained significant attention. Xenogeneic collagen matrices, in particular, have shown promising outcomes in terms of biocompatibility, angiogenesis, tissue integration, and reduced patient morbidity.

This review aims to provide a comprehensive overview of current soft tissue augmentation techniques, with emphasis on biologically driven scaffold materials and their clinical applications in periodontics and implantology.

## Introduction and background

The integrity and stability of peri-gingival and peri-implant soft tissues are fundamental determinants of long-term success in both periodontal and implant therapy [1]. Adequate dimensions of keratinized tissue and mucosal thickness are essential for maintaining optimal plaque control, minimizing inflammation, and preserving soft tissue architecture, thereby contributing to both functional and esthetic outcomes. Maintenance of healthy soft tissue architecture is therefore considered an essential prerequisite for the long-term preservation of both natural dentition and dental implants [2].

Despite similarities in clinical appearance, periodontal and peri-implant soft tissues differ considerably in their structural and biologic characteristics. Around natural teeth, the presence of the periodontal ligament (PDL) provides vascular supply, proprioception, and a firm connective tissue attachment, thereby offering greater resistance to mechanical and microbial challenges. In contrast, dental implants lack a PDL and instead depend on a peri-implant mucosal seal, which is structurally less vascularized and functionally more vulnerable to inflammatory breakdown. Consequently, peri-implant tissues are more susceptible to recession, marginal bone loss, and peri-implant inflammation, particularly in individuals exhibiting a thin soft tissue phenotype [2,3].

The soft tissue phenotype, previously referred to as tissue biotype, has therefore emerged as a critical factor influencing the stability and predictability of periodontal and implant therapy. Thin phenotypes are frequently associated with increased risk of soft tissue recession, compromised esthetic outcomes, and reduced long-term tissue stability. In response to these challenges, soft tissue augmentation procedures have become an integral component of contemporary periodontal and peri-implant therapy, aimed at increasing keratinized tissue width, enhancing mucosal thickness, and improving peri-implant tissue stability [3].

Historically, autogenous grafts such as free gingival grafts (FGG) and subepithelial connective tissue grafts (SCTG) have been regarded as the gold standard for soft tissue augmentation due to their high predictability, biocompatibility, and long-term stability. These techniques have demonstrated excellent outcomes in increasing keratinized tissue width, enhancing soft tissue thickness, and achieving root coverage. However, their use is associated with several limitations, including donor site morbidity, increased surgical time, postoperative discomfort, and limited availability of donor tissue, which may restrict their applicability in certain clinical scenarios [3-6].

In response to these limitations, there has been a paradigm shift toward the development and utilization of soft tissue substitutes (STSs). These biomaterials, including allogenic, xenogeneic, alloplastic, and tissue-engineered scaffolds [7], are designed to mimic the extracellular matrix, promote angiogenesis, and facilitate cellular migration and tissue integration. Among these, xenogeneic collagen matrices and acellular dermal matrices (ADMs) have gained considerable attention due to their favorable biological properties, reduced patient morbidity, and elimination of the need for a second surgical site. This evolution reflects a broader transition in periodontal therapy toward minimally invasive, patient-centered approaches that aim to achieve comparable clinical outcomes while improving patient comfort and acceptance [4].

Furthermore, advancements in tissue engineering and regenerative medicine have introduced bioactive and cell-based constructs that incorporate growth factors, stem cells, and scaffold technologies to enhance healing and regeneration [8]. These innovations aim not only to replace lost tissue but also to actively promote biologic regeneration and functional restoration. Despite these advances, clinical outcomes remain highly dependent on multiple factors, including defect characteristics, tissue phenotype, surgical technique, and patient-related variables [9].

Therefore, a comprehensive understanding of soft tissue biology, biomaterial properties, and clinical indications is essential for selecting appropriate augmentation strategies and achieving predictable, stable, and esthetically satisfactory outcomes in both periodontal and implant therapy.

## Review

The present manuscript was designed as a comprehensive narrative review intended to summarize contemporary evidence regarding STSs and augmentation procedures in periodontal and peri-implant therapy.

This narrative review was compiled through a comprehensive literature search performed using electronic databases, including PubMed/Medical Literature Analysis and Retrieval System Online (MEDLINE), Scopus, and Cochrane Library. Literature published primarily between 2000 and 2025 was screened using combinations of keywords including “soft tissue augmentation,” “periodontal plastic surgery,” “peri-implant mucosa,” “collagen matrix,” “connective tissue graft,” “keratinized mucosa,” “soft tissue substitutes,” and “tissue engineering.” Priority was given to randomized clinical trials, systematic reviews, consensus reports, landmark studies, and recent high-quality cohort studies relevant to periodontal and peri-implant soft tissue augmentation. Additional references were identified through manual screening of reference lists from selected articles.

The present manuscript was designed as a comprehensive narrative review of contemporary soft tissue augmentation approaches in periodontal and peri-implant therapy; therefore, no formal meta-analysis or quantitative evidence synthesis was performed.

The evolution of soft tissue augmentation in periodontal and implant therapy reflects a progressive transition from autogenous grafting techniques to the integration of advanced biomaterials. Since the seminal introduction of the autogenous FGG, the focus has been on increasing the width of attached gingiva to enhance peri-gingival tissue stability around natural teeth [6]. FGGs were initially employed to augment keratinized tissue and later adapted for root coverage in cases of gingival deformities.

As clinical goals expanded to include not only increased tissue dimensions but also improved esthetic outcomes, complete root coverage, and long-term durability, the SCTG, particularly when combined with a coronally advanced flap (CAF), emerged as the technique of choice. This combination remains the gold standard for the management of single and multiple mucogingival defects, offering highly predictable outcomes and superior aesthetic integration [3].

Despite its clinical efficacy, SCTG harvesting poses inherent disadvantages, including the need for a secondary surgical site, increased operative time, postoperative pain, and limited availability of donor tissue [9]. These drawbacks thus catalyzed the invention of less invasive alternatives with comparable clinical outcomes.

Beginning in the 1980s, STSs were introduced into mucogingival surgery as viable alternatives to autogenous grafts. These substitutes aimed to address the shortcomings of traditional techniques by eliminating donor site morbidity, reducing surgical time, and enhancing patient comfort [10]. Evidence suggests that with each additional minute of surgery, the incidence of moderate to severe postoperative pain and swelling increases by approximately 3%-4%, emphasizing the clinical importance of time efficiency [11].

STSs encompass a diverse group of biomaterials that have been divided based on their origin into allogenic, xenogeneic, alloplastic, and living constructs (cell-based scaffolds). Over time, the most frequently adopted matrices have included ADMs, porcine-derived collagen scaffolds (single, bilayer, and volume-stable forms), and polymeric matrices such as amniotic membranes. These biomaterials are designed to provide biocompatible scaffolding, support angiogenesis, and facilitate soft tissue integration without the need for autogenous tissue harvesting [5].

This transition from autogenous to biomaterial-based approaches represents a paradigm shift in modern mucogingival and peri-implant therapy, aligning with the broader movement toward minimally invasive and patient-centered surgical techniques. Continued innovation in scaffold design and biological enhancement is likely to further refine these alternatives and expand their clinical applications in both periodontal and implant dentistry (Table 1) [12].

**Table 1:** Evolution of soft tissue substitutes

Phase / Period	Technique or Material	Objective / Key Advancement	Advantages	Limitations / Drawbacks
Initial phase (1960s–1970s)	Autogenous free gingival graft [4]	To increase the width of attached gingiva and improve peri-gingival stability around natural teeth.	Predictable keratinized tissue gain; stable outcomes.	Poor aesthetics, color mismatch, and donor site morbidity.
Intermediate phase (1980s–1990s)	Subepithelial connective tissue graft with a coronally advanced flap [5]	To achieve root coverage, enhance aesthetics, and increase soft-tissue thickness. Became the “gold standard” for mucogingival defects.	Excellent aesthetics; high predictability; durable outcomes.	Requires secondary surgical site, limited donor tissue, increased operative time, and postoperative discomfort.
Transitional phase (1990s–2000s)	Introduction of soft tissue substitutes [6]	To eliminate donor site morbidity and reduce surgical time while maintaining comparable results to autografts.	Minimally invasive; improved patient comfort; reduced operative duration.	Variable outcomes depending on material type and technique sensitivity.
Contemporary phase (2000s–Present)	Biomimetic matrices and scaffolds (allogenic, xenogeneic, alloplastic, and living constructs), e.g., acellular dermal matrix, porcine collagen scaffolds (Mucograft®, Fibro-Gide®), amniotic membrane [3]	To provide biocompatible scaffolds supporting angiogenesis, cellular infiltration, and soft-tissue regeneration without autogenous grafting.	No donor site; good integration; aesthetically pleasing; reduced patient morbidity.	Long-term data still evolving; higher material cost.

Understanding these advances requires an appreciation of the biological differences between periodontal and peri-implant soft tissues.

Structural and histological differences between peri-implant and natural tooth tissues

The gingival epithelium surrounding natural teeth is predominantly keratinized and transitions apically into the junctional epithelium (JE), which attaches to the enamel surface via hemidesmosomes along its entire length. Beneath this epithelial layer, the connective tissue and PDL form a highly specialized and dynamic support system. The PDL, with an average width of 0.2-0.3 mm, contains well-organized collagen fiber bundles that insert perpendicularly into both cementum and alveolar bone, thereby providing mechanical stability and acting as a biologic barrier against microbial invasion [13].

In contrast, peri-implant tissues lack a PDL and instead rely on a peri-implant mucosal seal for protection. The peri-implant mucosa consists of a keratinized epithelial layer and an underlying connective tissue that is in direct contact with the implant surface. The epithelial component forms a JE-like structure, often referred to as the peri-implant epithelium, which typically extends approximately 2 mm apical to the mucosal margin and contributes to the formation of a biological barrier [13].

The connective tissue surrounding dental implants exhibits distinct structural characteristics compared to that of natural teeth. It is composed of an inner fiber-rich zone adjacent to the implant surface, measuring approximately 50 µm in thickness, and an outer vascular zone. Unlike the perpendicular fiber orientation observed around natural teeth, collagen fibers in peri-implant tissues are predominantly oriented parallel or circumferentially to the implant surface and do not insert into the implant. This lack of fiber insertion reduces the mechanical anchorage and resistance to apical epithelial migration [14].

Furthermore, the biologic width around natural teeth averages approximately 2 mm, comprising nearly equal contributions from the JE and connective tissue attachment. In contrast, the peri-implant biologic width is generally greater, ranging from approximately 3 to 4 mm, with a longer epithelial component and a relatively shorter connective tissue zone. The overall thickness of peri-implant mucosa may vary depending on clinical conditions and tissue phenotype [14].

In addition to structural differences, peri-implant tissues demonstrate reduced vascularity due to the absence of the PDL and its associated vascular network. This diminished blood supply may compromise immune surveillance and wound healing capacity. Consequently, peri-implant tissues are more susceptible to inflammation and breakdown under microbial challenge.

Collectively, these structural and histological differences highlight the comparatively less robust nature of the peri-implant soft tissue seal and underscore the importance of optimizing soft tissue conditions to enhance long-term stability and clinical outcomes [15].

These structural differences directly influence the healing dynamics and biological behavior of peri-implant tissues.

Mechanisms and phases of soft tissue healing after augmentation procedures

Oral soft tissue wound healing follows a well-orchestrated biological sequence involving hemostasis, inflammation, proliferation, and remodeling. Despite this consistent pattern, the phenotypic characteristics of gingival, alveolar, and palatal mucosa are largely governed by their intrinsic biological properties rather than functional adaptation. Notably, granulation tissue originating from the PDL or from connective tissue previously associated with keratinized epithelium has demonstrated the capacity to induce keratinization. In contrast, deeper palatal connective tissue exhibits a comparatively limited potential to promote keratinized tissue formation when compared to the superficial subepithelial connective tissue [16].

Epithelial healing following both surgical and non-surgical periodontal procedures is generally completed within seven to 14 days. During this period, initial adhesion and structural integrity between the soft tissue flap and the root surface are established, typically by the end of the second postoperative week. However, in the context of dental implants, soft tissue maturation follows a delayed timeline. The establishment of the peri-implant biological width and functional mucosal seal usually requires approximately six to eight weeks [16].

These variations in healing dynamics are closely related to the distinct structural characteristics of peri-implant tissues. Unlike the periodontium of natural teeth, the connective tissue surrounding implants resembles scar-like tissue, characterized by reduced vascularity, altered collagen fiber orientation (primarily parallel to the implant surface), and differences in cellular composition. Additionally, the peri-implant JE may exhibit increased apical extension, particularly in implants placed immediately into extraction sockets compared to those placed in healed ridges. These structural differences significantly influence the stability, integration, and long-term outcomes of soft tissue augmentation procedures around implants [15].

These biological principles form the foundation for the selection and application of biomaterials and surgical techniques in soft tissue augmentation.

Biomaterials and extracellular matrices for soft tissue augmentation

Scaffolds are critical biomaterials in tissue engineering and regenerative medicine, providing a three-dimensional porous matrix that supports cell adhesion, proliferation, extracellular matrix deposition, and ultimately tissue regeneration. Effective scaffolds facilitate nutrient and gas exchange, degrade in harmony with tissue repair, and minimize adverse host responses (Table 2).

**Table 2:** Generations of soft tissue matrices Source: [17]

Generation	Type of Graft/Material	Source	Key Characteristics	Examples
First generation	Autogenous grafts	Patient (intraoral donor site)	Gold standard; excellent biocompatibility; contains living cells; requires a second surgical site	Free gingival graft, subepithelial connective tissue graft
Second generation	Acellular dermal matrices (allografts)	Human donor tissue (processed)	Cell-free scaffold; avoids donor site morbidity; good integration; variable thickness	Acellular dermal matrix, e.g., AlloDerm
Third generation	Xenogeneic collagen matrices	Animal-derived (mainly porcine)	Off-the-shelf availability; no second surgical site; acts as a scaffold; variable long-term stability	Mucograft® collagen matrix
Fourth generation	Tissue-engineered/bioactive matrices	Synthetic/biologically enhanced constructs	Incorporates biologics; promotes regeneration; enhances healing and angiogenesis	Platelet-derived growth factor, bone morphogenetic protein, platelet-rich fibrin, stem cell-based constructs

Human-Derived Scaffolds

Human-derived scaffolds such as ADMs and human amniotic membranes (HAM) have been extensively investigated as alternatives to autogenous grafts for periodontal and peri-implant soft tissue augmentation.

ADMs are derived from donated human skin after removal of all cellular components, providing a biocompatible scaffold ideal for healing of the soft tissues. These matrices create a favorable setting for neovascularization and colonization by the patient's own cells, making them an effective solution for soft tissue augmentation and periodontal regenerative procedures. A key advantage of using ADMs is the elimination of a secondary surgical site, such as the palate, which helps minimize patient discomfort and reduces operative time. However, they have certain drawbacks, such as postoperative shrinkage and limited ability to reliably promote keratinized tissue formation, which may contribute to partial relapse of the treated site [18]. 

Studies have shown that ADMs integrate effectively with surrounding oral tissues and promote healing by supporting the migration of fibroblasts and keratinocytes through the release of native growth factors. Notably, even when only fibroblasts are present, keratinization of the epithelium still occurs successfully, indicating that these grafts maintain their functional integrity. Due to their close color resemblance to natural gingiva, high biocompatibility, and ability to enhance soft tissue thickness, ADMs are increasingly favored for gingival reconstruction and contouring around both natural teeth and dental implants [19].

Conversely, HAMs represent another type of biologically derived graft material. These thin membranes are made up of three layers: a surface epithelial layer, a strong basement membrane, and a collagen-rich middle layer that lacks blood vessels. They are naturally loaded with powerful growth factors such as epidermal growth factor, transforming growth factor-α, transforming growth factor-β, fibroblast growth factor-2, and keratinocyte growth factor. These molecules give HAM its unique healing properties, including the ability to reduce inflammation, combat infections, relieve pain, prevent scar tissue formation, and promote epithelial healing, collagen growth, and new blood vessel formation, all key for success in periodontal and peri-implant procedures [19].

Also, they are delicate and can be difficult to handle in surgery, and they tend to degrade quickly once placed in the body. Moreover, since both ADM and HAM are derived from human tissue, their use raises ethical concerns and carries a small, theoretical risk of disease transmission, which is an important consideration when planning treatment and discussing options with patients [3].

Overall, human-derived scaffolds represent effective minimally invasive alternatives for selected clinical situations, particularly when reduction of patient morbidity and improved aesthetic integration are prioritized.

Animal-Derived Scaffolds

**Figure 1:**
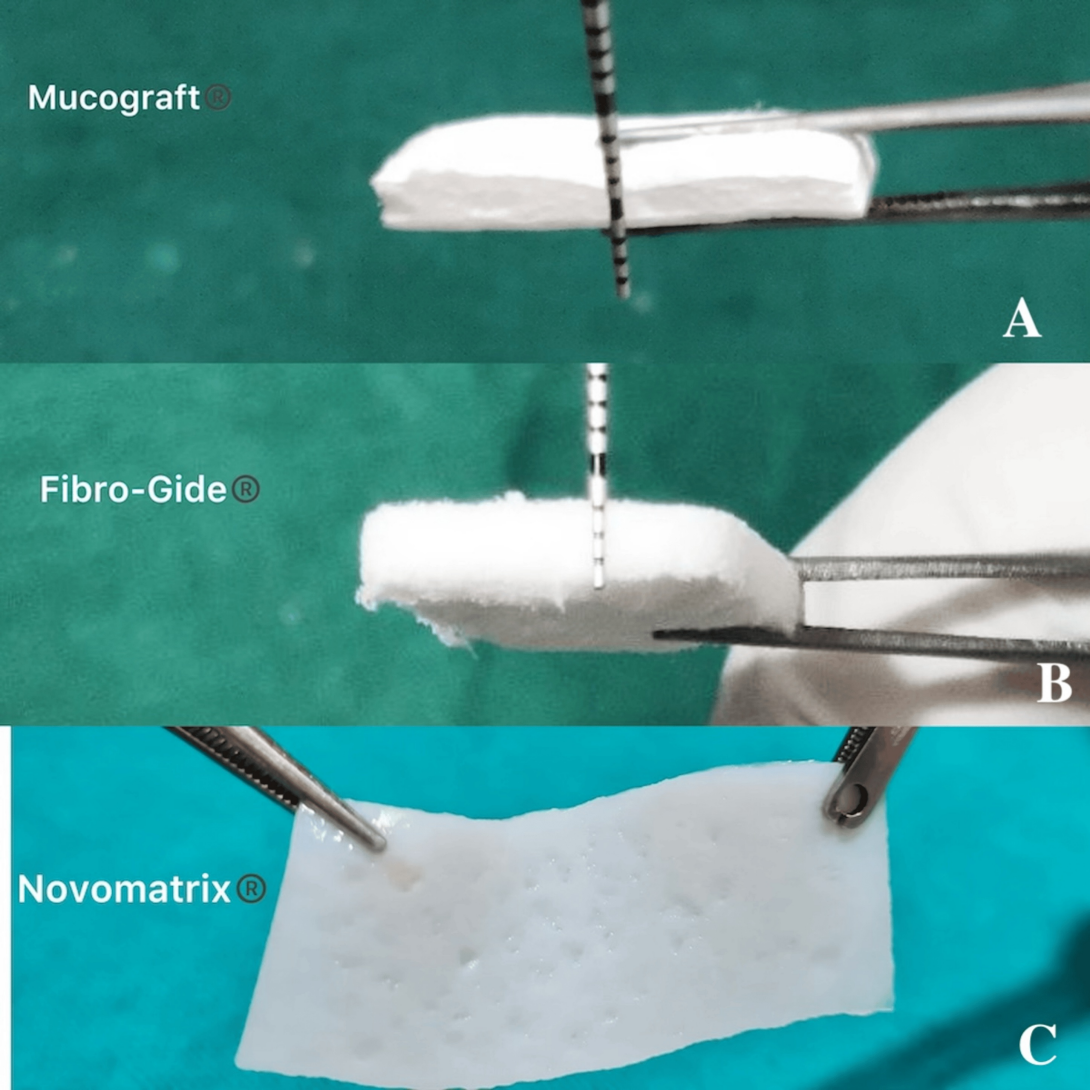
Structural characteristics of different xenogeneic collagen matrices used for soft tissue augmentation A. Mucograft®; B. Fibro-Gide®; C. Novomatrix® Images photographed by the authors.

**Figure 2:**
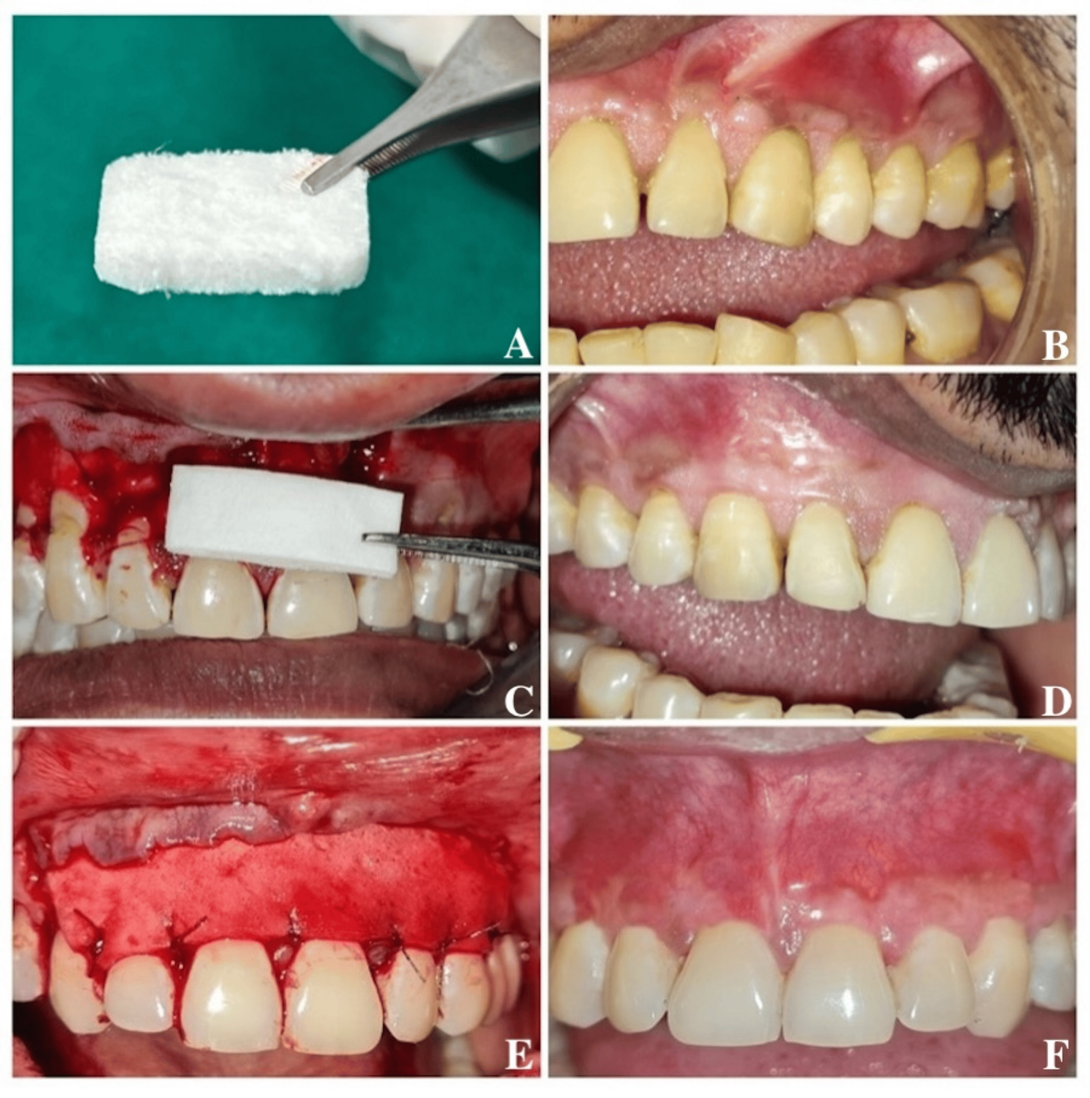
Clinical placement of a collagen-based soft tissue substitute for mucogingival augmentation A. Fibro-Gide®; B. Volumetric change in soft tissue after the placement of Fibro-Gide®; C. Mucograft®l D. Change in soft tissue after the placement of Mucograft®; E. Novomatrix®; F. Change in soft tissue after the placement of Novomatrix® The images are of the authors’ patients and are published with signed informed consent.

Animal-derived scaffolds, particularly xenogeneic collagen matrices, have gained popularity in periodontal and peri-implant soft tissue augmentation as alternatives to autogenous grafts. Most commercially available matrices are porcine-derived dermal matrices. These xenogeneic collagen-based materials provide several advantages, such as greater availability, reduced cost, and ease of procurement in large quantities. They are primarily composed of type I and type III collagen (Figures 1, 2). They are designed to support tissue reconstruction and facilitate the development of neo-connective tissue within the scaffold.

These materials are characterized by various biological advantages: they degrade naturally without provoking a foreign body response, integrate well with host tissue, promote rapid vascularization, exhibit hemostatic and fibroblast-attracting properties, and support osteoblast adhesion. Their weak immunogenicity, biocompatibility, and ability to assist in soft tissue healing make them highly suitable for periodontal and peri-implant soft tissue reconstruction. Degradation of these matrices occurs through enzymatic pathways, including collagenases, bacterial proteases, and enzymes secreted by immune cells like macrophages and polymorphonuclear leukocytes [5]. 

**Figure 3:**
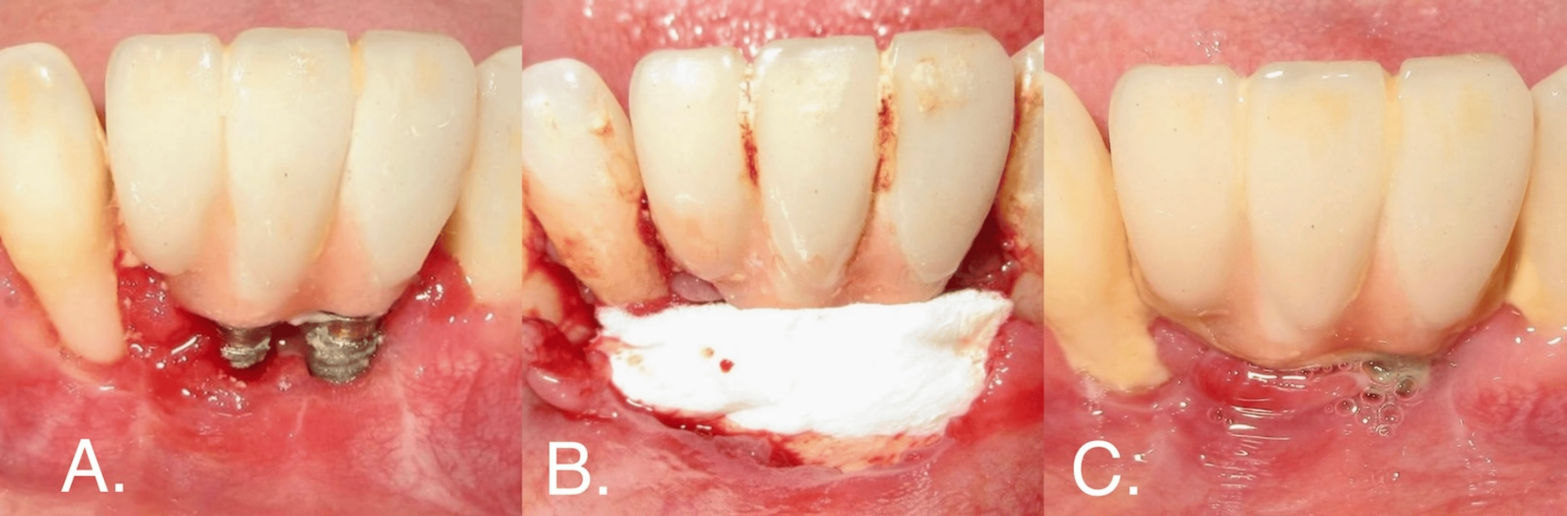
Soft tissue augmentation with Mucograft® around implants A. Peri-implant soft tissue dehiscence; B. Mucograft® in-situ; C. Change in soft tissue after three months The images are of the authors’ patients and are published with signed informed consent.

Mucograft® (Geistlich Pharma AG, Wolhusen, Switzerland) is a widely used porcine-derived, non-cross-linked, resorbable collagen matrix composed of type I and III collagen. It features a bilayered design, with a dense, smooth collagen layer that promotes cell adhesion and a porous layer oriented toward the host tissue to enhance integration, tissue regeneration, and angiogenesis. The scaffold’s porous architecture plays a critical role in vascularization, an essential factor in successful tissue engineering, since oxygen diffusion can only adequately reach cells within approximately 100-200 µm of a blood vessel. While Mucograft® is capable of increasing the width of keratinized tissue, its lack of native cellular components has raised concerns regarding its ability to support full keratinization. Nevertheless, it has shown promise as a supportive matrix for the proliferation of fibroblasts and spherical keratinocytes, making it a viable alternative for soft tissue regeneration. On the initiation of the procedure, when Mucograft® is placed at a surgical site, fibroblasts begin producing key biomolecules like fibronectin, cytokines, glycosaminoglycans, and human dermal collagen. These components help create a metabolically active matrix that supports soft tissue healing. This matrix not only promotes the migration and attachment of nearby cells but also plays a pivotal role in wound closure through re-epithelialization and angiogenesis. Additionally, it encourages keratinocyte migration and adhesion, further aiding in regeneration (Figure 3) [5].

Following the placement of such tissue-engineered materials, a range of angiogenesis-related biomarkers is expressed. These include growth factors and signaling proteins like platelet-derived growth factor-BB, vascular endothelial growth factor, angiogenin, angiostatin, fibroblast growth factor-2, interleukin-8, and granulocyte-macrophage colony-stimulating factor, as well as tissue remodeling regulators such as tissue inhibitors of metalloproteinases- 1,2. and interferon gamma-induced protein. Together, these mediators enhance vascularization and accelerate the wound healing process [20]. 

Mucoderm® (Botiss GmbH, Zossen, Germany) is a porcine-derived ADM that undergoes a meticulous multi-step purification process to eliminate all antigenic components while conserving the native architecture of the extracellular collagen matrix. This intact three-dimensional structure provides an ideal scaffold for reattachment and growth of fibroblasts and endothelial cells, thereby supporting efficient tissue integration and rapid revascularization at the recipient site [5].

The performance of 3D matrices like porcine ADM is influenced by surface modifiers such as porous texture and interconnectivity, which facilitate the infiltration and growth of human fibroblasts, endothelial cells, osteoblasts, and keratinocytes, which are essential for integration with surrounding tissues. Mucoderm® serves as a biocompatible scaffold that supports keratinocyte attachment, growth, and differentiation, mimicking the natural extracellular matrix. Dermal fibroblasts regulate matrix homeostasis and interact with keratinocytes through paracrine signaling, promoting soft tissue healing via growth factors such as fibroblast growth factor-2, platelet-derived growth factor-BB, bone morphogenetic proteins, insulin-like growth factor-1, and vascular endothelial growth factor. Successful matrix incorporation depends on microvascular ingrowth through angiogenesis, ensuring perfusion and preventing avascular wound complications. Additionally, Mucoderm® is utilized as a source of enamel matrix derivatives in treating gingival deformities [5]. 

Fibro-Gide® (Geistlich Pharma AG, Wolhusen, Switzerland) offers excellent tissue volume stability due to its cross-linked collagen structure, which enhances mechanical strength and slows degradation. Cross-linking methods, physical (e.g., UV, gamma irradiation, and heat) and biological (e.g., transglutaminase), improve tensile strength but may generate degradation byproducts with potential toxicity. Additionally, cross-linked collagen membranes tend to delay angiogenesis as compared to the non-cross-linked membranes. Fibro-Gide’s porous layer supports fibroblast infiltration, integration of tissue, and matrix production, though it requires adequate healing for optimal results (Figure 2) [5]. 

DynaMatrix® (Keystone Dental, Burlington, MA, USA) is an elastic extracellular matrix scaffold that supports fibroblast, epithelial, and blood vessel growth, quickly regaining its volume after placement. Both collagen-derived matrices and connective tissue grafts undergo significant volume loss during the first month due to biodegradation, followed by replacement with new connective tissue. Cross-linking of collagen matrices can slow degradation and improve tissue formation, but may trigger inflammatory responses, compromising healing [5].

Soft tissue augmentation involving cellular therapy

Recent advances in tissue engineering have introduced cellular therapy-based approaches for periodontal and peri-implant soft tissue augmentation. These strategies utilize bioengineered constructs containing fibroblasts, keratinocytes, stem cells, or biologically active scaffolds to enhance wound healing and tissue regeneration [21].

Living cellular constructs and platelet concentrates such as platelet-rich fibrin (PRF) have demonstrated promising results in promoting angiogenesis, soft tissue maturation, and aesthetic integration while reducing donor-site morbidity [22,23]. Similarly, stem cell-based therapies and bioactive scaffolds are being investigated for their regenerative potential in periodontal and peri-implant reconstruction.

Despite encouraging early outcomes, current evidence remains limited because of variability in study design, biomaterials, and follow-up duration. Consequently, autogenous connective tissue grafts continue to represent the gold standard for predictable soft tissue augmentation, particularly in complex clinical situations [11].

Overall, cellular therapy-based approaches represent a promising emerging field; however, further long-term randomized clinical trials are required before routine clinical application can be recommended [18].

Indications for soft tissue augmentation around natural teeth

Soft tissue augmentation around natural teeth is performed to improve periodontal stability, enhance aesthetics, and facilitate patient comfort during oral hygiene procedures. Although periodontal health may be maintained in areas with minimal keratinized tissue when plaque control is adequate, certain clinical situations still benefit significantly from augmentation procedures [24, 25].

Thin gingival phenotypes are particularly susceptible to gingival recession, marginal tissue breakdown, and aesthetic compromise, especially in the presence of subgingival restorations or traumatic tooth brushing [26]. Similarly, orthodontic tooth movement beyond the alveolar housing may predispose to alveolar dehiscence and soft tissue recession, thereby increasing the need for phenotype modification and tissue thickening procedures [27].

Soft tissue augmentation is also indicated in areas with inadequate keratinized tissue, high frenum attachment, shallow vestibular depth, root hypersensitivity associated with recession defects, and patient discomfort during plaque control measures [24]. In such cases, augmentation procedures aim to improve tissue thickness, enhance plaque control, increase patient comfort, and promote long-term periodontal stability. From an aesthetic perspective, root coverage procedures are frequently performed to correct gingival recession defects and improve smile harmony.

A CAF combined with a connective tissue graft remains the gold standard for treatment of isolated recession defects because of its high predictability and favorable aesthetic outcomes [28].

Overall, treatment selection should be based on tissue phenotype, recession classification, aesthetic demands, and patient-specific risk factors to achieve predictable and stable long-term outcomes.

A summary of the commonly used STSs, their sources, advantages, limitations, and principal clinical indications is presented in Table 3.

**Table 3:** Classification and clinical characteristics of commonly used soft tissue substitutes and grafting materials in periodontal and peri-implant soft tissue augmentation.

Biomaterial/Scaffold	Source	Advantages	Limitations	Clinical Indications
Free gingival graft	Autogenous	Predictable increase in keratinized tissue width; long-term stability	Donor site morbidity, poor color match, and postoperative discomfort	Increasing keratinized tissue width around teeth and implants
Subepithelial connective tissue graft	Autogenous	Gold standard for root coverage and tissue thickening; excellent vascularity and aesthetics	Second surgical site required; limited tissue availability	Root coverage, phenotype modification, peri-implant soft tissue augmentation
Acellular dermal matrix	Human-derived allograft	Eliminates donor site morbidity; good aesthetic integration; reduced surgical time	Graft shrinkage; variable keratinized tissue gain	Root coverage, soft tissue thickening, peri-implant soft tissue augmentation
Human amniotic membrane	Human-derived allograft	Anti-inflammatory and proangiogenic properties; enhanced wound healing	Rapid degradation; delicate handling; limited long-term evidence	Soft tissue healing and regenerative procedures
Mucograft®	Porcine-derived collagen matrix	Reduced morbidity; favorable angiogenesis and tissue integration	Less predictable volumetric stability compared with connective tissue graft	Keratinized tissue augmentation and peri-implant soft tissue enhancement
Fibro-Gide®	Cross-linked porcine collagen matrix	Improved volumetric stability and soft tissue thickness gain	Cost; excessive cross-linking may impair vascularization	Peri-implant soft tissue volume augmentation
Mucoderm®	Porcine-derived collagen matrix	Good biocompatibility and handling characteristics	Limited long-term comparative evidence	Soft tissue thickening and peri-implant phenotype modification
Platelet-rich fibrin	Autologous platelet concentrate	Growth factor release; enhanced wound healing and angiogenesis	Variable preparation protocols; limited volumetric stability	Adjunctive soft tissue healing and regenerative procedures
Living cellular constructs	Tissue-engineered cellular scaffold	Reduced donor morbidity; favorable aesthetic integration	High cost; limited long-term evidence	Keratinized tissue augmentation and regenerative therapy
Stem cell/Bioactive scaffolds	Tissue-engineered regenerative constructs	Potential for enhanced regeneration and tissue remodeling	Experimental nature; lack of standardized protocols	Emerging regenerative periodontal and peri-implant therapy

Indications for soft tissue augmentation around implants

Soft tissue augmentation around dental implants is indicated in several clinical situations where inadequate tissue quality or quantity may compromise peri-implant health and aesthetic outcomes. A thin gingival phenotype is a common indication, as it is associated with an increased risk of mucosal recession and implant show-through, particularly in the aesthetic zone. Similarly, insufficient keratinized mucosa, typically defined as less than 2 mm, has been linked to plaque accumulation, patient discomfort during oral hygiene procedures, and a higher susceptibility to peri-implant inflammation [3].

Peri-implant mucosal recession represents another important indication, as it can result in the exposure of implant components, leading to both functional and aesthetic concerns. In addition, soft tissue augmentation is often required in cases with aesthetic demands or tissue deficiencies, including soft tissue dehiscence, inadequate tissue volume, or when site development and contour optimization are necessary to achieve harmonious peri-implant architecture. In such scenarios, augmentation procedures aim to enhance tissue thickness, improve stability, and ensure long-term peri-implant health and aesthetic integration [15].

Surgical considerations for soft tissue augmentation

Soft tissue augmentation procedures can broadly be categorized into two primary clinical approaches: (1) techniques aiming at increasing the keratinized mucosal width; (2) those focused on enhancing the dimensions or volume of the peri-implant and peri-gingival structures.

Among the various parameters influencing long-term aesthetic and functional outcomes, mucosal thickness has emerged as a critical determinant of peri-implant and periodontal stability.

Numerous studies have demonstrated that the presence of an adequate keratinized mucosal zone is positively linked with enhanced soft and hard tissue preservation, improved plaque control, and reduced risk of mucosal recession around both natural teeth and dental implants. In contrast, insufficient keratinized mucosa has been linked to increased peri-implant inflammation, compromised oral hygiene, and progressive soft tissue breakdown.

Clinical cohort data indicate that suturing technique (for example, horizontal apical mattress sutures) can measurably influence postoperative keratinized mucosa width and thickness and should be considered when comparing surgical protocols [29].

According to Lang & Loe, a minimum of 2 mm of keratinized tissue (with ≥1 mm attached gingiva) is sufficient in the maintenance of periodontal health. Clinically, a minimum of 2 mm of keratinized mucosa is recommended to maintain peri-implant health and ensure functional and aesthetic integration, mirroring the standard also suggested for natural dentition [24]. In addition to width, mucosal thickness plays a pivotal role in aesthetic outcomes, particularly in the anterior region. When the mucosal thickness exceeds 2 mm, it significantly limits the optical translucency of the soft tissue, thereby masking the underlying restorative components such as zirconia or titanium abutments. This helps achieve a more natural appearance by reducing the risk of color shine-through, which is critical in highly aesthetic zones [3].

According to Wennstrom, attached gingiva is not mandatory with good plaque control but critical in suboptimal hygiene conditions. Together, adequate width of keratinized mucosa and sufficient thickness of alveolar mucosa contribute synergistically to the maintenance of soft tissue architecture, aesthetic harmony, and the appreciable clinical outcomes of periodontal and peri-implant therapies [25].

Based on these biological considerations, soft tissue augmentation techniques are selected according to defect characteristics and site-specific requirements.

Soft tissue augmentation around natural tooth

Gingival augmentation procedures are primarily aimed at increasing the width of keratinized tissue and enhancing gingival thickness to improve both functional and aesthetic outcomes. These procedures can be broadly classified into two categories: (1) non-root coverage procedures and (2) root coverage procedures.

Non-root Coverage Procedures

Non-root coverage procedures are primarily indicated to enhance patient comfort during oral hygiene practices, improve plaque control, and prevent the progression of gingival recession in susceptible areas. Although the necessity of a specific width of keratinized tissue remains a subject of debate, most clinicians agree that at least 1 mm of attached gingiva is adequate in patients with good oral hygiene and a favorable periodontal condition.

Soft tissue augmentation around natural teeth is primarily performed to increase keratinized tissue width, enhance gingival thickness, and achieve root coverage in areas affected by gingival recession. These procedures aim to improve periodontal stability, reduce dentinal hypersensitivity, facilitate plaque control, and enhance aesthetic outcomes [11].

**Figure 4:**
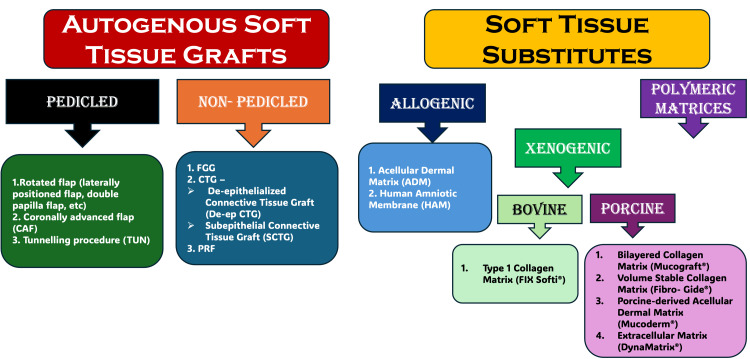
Classification of soft tissue grafts based on their origin Source: This figure is an original illustration created by the authors using Microsoft PowerPoint (Microsoft Corp., Redmond, WA, USA).

Grafting techniques in periodontal plastic surgery are showcased in Figure 4.

Grafting procedures demonstrate high predictability due to the preservation of intrinsic biological characteristics of the transplanted tissue. Based on vascular supply, grafts can be classified as (1) pedicle grafts: maintain a vascular connection with the donor site; (2) free grafts: harvested from a donor site and transferred to a recipient site.

Among free grafts, the FGG was introduced to increase keratinized tissue width. While effective for this purpose, FGG is associated with limitations such as compromised vascularization, poor color match, and reduced predictability in achieving complete root coverage [6].

The SCTG represents a major advancement in periodontal plastic surgery and is widely regarded as the gold standard for soft tissue augmentation. A connective tissue graft enhances soft tissue thickness, improves vascular integration, and provides superior aesthetic outcomes. It has been extensively used for root coverage, peri-implant soft tissue enhancement, and papillary reconstruction [30].

Zucchelli et al. described a connective tissue graft as a biological modifier that enhances flap stability and adaptation during early healing, thereby improving tissue thickness and predictability of root coverage outcomes. An ideal biomaterial for soft tissue augmentation should exhibit long-term volume stability and favorable biological behavior to support tissue remodeling [31].

Despite its clinical advantages, a connective tissue graft is associated with limitations such as increased surgical time, donor site morbidity, and limited tissue availability. Consequently, alternative materials, including collagen-based dermal matrices and other STSs, have been introduced to reduce patient morbidity while maintaining acceptable clinical outcomes.

Minimally invasive approaches such as the modified coronally advanced tunnel (MCAT) and vestibular incision subperiosteal tunnel access (VISTA) techniques have also gained popularity because they preserve vascularity, minimize surgical trauma, and improve aesthetic outcomes [32]. In addition, collagen matrices and other STSs have been investigated as alternatives to autogenous grafts to reduce patient morbidity, although their long-term predictability may remain inferior to connective tissue grafts [32].

Treatment selection should therefore be based on recession classification, tissue phenotype, aesthetic demands, and patient-related considerations to achieve stable and predictable clinical outcomes.

Root Coverage Procedures

**Figure 5:**
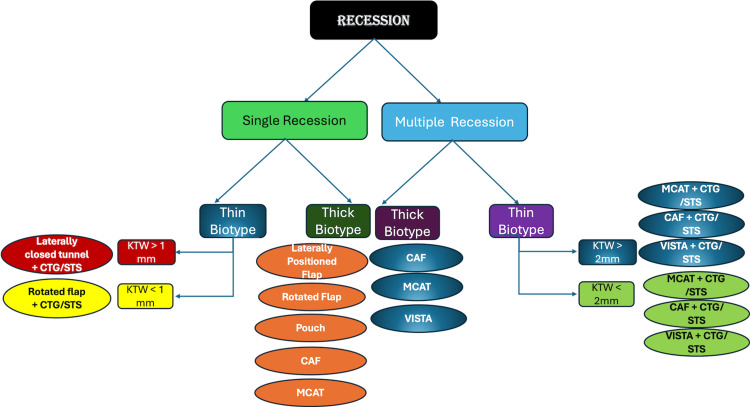
Decision-tree for treatment of gingival recession CAF: coronally advanced flap; CTG: connective tissue graft; FGG: free gingival graft; KTW: keratinized tissue width; MCAT: modified coronally advanced tunnel; VISTA: vestibular incision subperiosteal tunnel access; STS: soft tissue substitutes Source: This figure is an original illustration created by the authors using Microsoft PowerPoint (Microsoft Corp., Redmond, WA, USA).

Root coverage procedures are outlined in Figure 5.

Root coverage procedures are primarily performed to treat gingival recession defects, restore soft tissue architecture, and achieve stable periodontal conditions with shallow probing depths.

In natural dentition, gingival recession is classified according to the 2017 World Workshop (Cairo classification) into RT1, RT2, and RT3 defects, based on the extent of interproximal attachment loss. This classification is clinically significant as it directly influences treatment selection and the predictability of root coverage outcomes [33].

RT1 defects, characterized by the absence of interproximal attachment loss, present the most favorable prognosis. In such cases, the CAF combined with CTG remains the gold standard, providing highly predictable and aesthetic outcomes. Minimally invasive techniques, such as the MCAT combined with CTG, have demonstrated comparable efficacy with improved vascular preservation and reduced surgical trauma.

RT2 defects, where interproximal attachment loss does not exceed buccal attachment loss, exhibit reduced predictability. However, CAF combined with CTG continues to represent the most reliable treatment modality. Tunnel-based techniques may be considered in aesthetically demanding regions.

RT3 defects, characterized by greater interproximal attachment loss than buccal loss, have limited potential for complete root coverage. In such cases, treatment focuses on soft tissue augmentation using CTG, FGG, or biomaterial substitutes to improve tissue thickness, stability, and aesthetic appearance rather than achieving complete root coverage [34].

Soft tissue augmentation around implants

STSs for the Treatment of Peri-implant Soft Tissue Dehiscence

Soft tissue augmentation around dental implants is performed to improve peri-implant tissue stability, enhance aesthetic integration, and minimize the risk of mucosal recession and peri-implant inflammation. Particular attention is given to increasing keratinized mucosa width and mucosal thickness, especially in the aesthetic zone and in patients with a thin tissue phenotype [35].

Connective tissue grafts remain the gold standard for peri-implant soft tissue augmentation because of their superior long-term stability and predictable enhancement of mucosal thickness [9]. Connective tissue grafts are frequently utilized in conjunction with CAF, tunnel techniques, or implant site development procedures to improve peri-implant soft tissue contours and aesthetic outcomes.

STSs such as collagen matrices and ADMs have increasingly been investigated as alternatives to autogenous grafts to reduce patient morbidity and surgical time [7]. Xenogeneic collagen matrices have demonstrated favorable outcomes in increasing keratinized mucosa and soft tissue thickness, although complete defect coverage and long-term volumetric stability may remain less predictable compared with CTGs [36].

Peri-implant soft tissue dehiscence defects are commonly managed according to defect severity, implant position, and tissue phenotype. Minimally invasive approaches such as tunnel techniques and VISTA procedures may improve vascularity and reduce surgical trauma while enhancing soft tissue thickness and esthetic integration [31].

Although STSs offer promising clinical outcomes with reduced morbidity, current evidence continues to support CTGs as the most predictable treatment modality for complex peri-implant soft tissue deficiencies. Therefore, treatment selection should be individualized according to defect morphology, aesthetic demands, and patient-specific factors [9].

Zucchelli et al. (2019) classification of peri-implant soft tissue dehiscence provides a practical framework for diagnosis and treatment planning based on soft tissue margin position, implant positioning, and papillary height [31].

Class I peri-implant soft tissue dehiscence defects primarily require phenotype modification and are effectively managed using connective tissue grafts or collagen matrices, often via minimally invasive tunnel techniques such as MCAT or VISTA to enhance vascularity and reduce morbidity. In Class II defects, characterized by buccal recession with correct implant positioning, CAF combined with CTG remains the gold standard, although tunnel approaches and STSs may be used in aesthetically demanding cases.

Class III defects, associated with implant malposition, demonstrate limited predictability, with treatment mainly focused on soft tissue thickening using CTG, FGG, or substitutes to improve aesthetics rather than achieve complete coverage. Class IV defects present the poorest prognosis, often requiring partial augmentation with prosthetic compensation or, in advanced cases, implant replacement.

**Figure 6:**
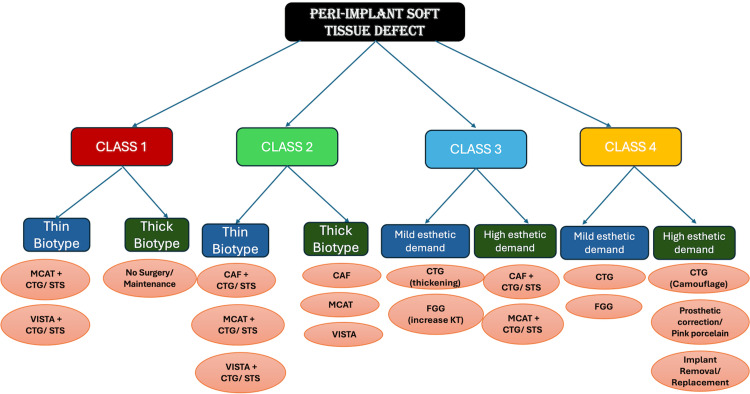
Decision-tree for treatment of peri-implant soft tissue dehiscence CAF: coronally advanced flap; CTG: connective tissue graft; FGG: free gingival graft; KT: keratinized tissue; MCAT: modified coronally advanced tunnel; VISTA: vestibular incision subperiosteal tunnel access; STS: soft tissue substitutes Source: This figure is an original illustration created by the authors using Microsoft PowerPoint (Microsoft Corp., Redmond, WA, USA).

Overall, CAF and tunnel-based approaches are the most versatile techniques for peri-implant soft tissue augmentation. While autogenous grafts remain the gold standard, STSs offer reduced morbidity and acceptable clinical outcomes, although complete defect coverage remains less predictable. Clinical success is strongly influenced by defect classification, implant positioning, and peri-implant phenotype; therefore, a classification-driven and individualized approach is essential for achieving stable and aesthetic outcomes (Figure 6) [37].

Diagnostic and clinical assessment of outcomes

The assessment of treatment outcomes following periodontal and peri-implant soft tissue augmentation procedures has traditionally been based on a combination of clinical parameters, radiographic evaluation, aesthetic indices, and both clinician- and patient-reported outcome measures. Commonly recorded clinical parameters include probing pocket depth, keratinized tissue width, gingival recession or peri-implant soft tissue dehiscence, and bleeding or suppuration on probing, typically assessed using a periodontal probe.

In addition to these conventional approaches, recent advances in diagnostic methodologies have enabled a more comprehensive evaluation of therapeutic outcomes, including changes in soft and hard tissue phenotype, as well as profilometric and volumetric alterations. The periodontal probe remains a widely utilized and practical tool not only for routine clinical measurements but also for the assessment of soft tissue phenotype. Conventionally, tissue phenotype is categorized as thin or thick based on probe transparency, whereas the use of color-coded probes allows for a more refined classification into thin, medium, thick, and very thick phenotypes [38].

Gingival thickness and mucosal thickness are commonly measured at standardized reference points, typically 1.5 mm and/or 3 mm apical to the soft tissue margin, using transgingival or transmucosal probing techniques. This method involves penetration of the soft tissue with an anesthetic needle or endodontic file, followed by measurement using a silicone stopper and digital calliper. Although simple and cost-effective, this technique is limited by potential inaccuracies related to instrument bending, displacement of the stopper, operator variability, and patient discomfort associated with its invasive nature [39].

To address these limitations, imaging modalities such as cone-beam computed tomography (CBCT) and digital intraoral scanning have gained increasing relevance. CBCT, particularly when combined with surface data obtained from digital impressions (STL files), enables a more precise assessment of both hard and soft tissue dimensions. Intraoral scanning, on the other hand, facilitates the longitudinal evaluation of volumetric changes following augmentation procedures; however, it necessitates multiple time-point recordings and, when used alone, provides limited information on soft tissue thickness unless integrated with CBCT data [40].

More recently, high-frequency ultrasonography (HFUS) has emerged as a promising, non-invasive, and radiation-free imaging modality for the evaluation of periodontal and peri-implant tissues [39]. HFUS enables real-time assessment of soft tissue thickness, underlying bone morphology, and vascular characteristics, thereby allowing differentiation between soft and hard tissue changes following augmentation procedures. Given the inherent limitations of transgingival probing, CBCT, and intraoral scanning, HFUS has been increasingly advocated as a reliable tool for assessing gingival and mucosal thickness, as well as buccal bone dimensions [41].

In addition, Doppler-based ultrasonography facilitates the evaluation of tissue perfusion by detecting frequency shifts generated by moving erythrocytes, thereby enabling visualization of blood flow within the tissues. This technique has demonstrated utility in distinguishing healthy from diseased peri-implant sites and in monitoring flap and graft vascularization during the healing phase [42].

Furthermore, strain elastography, an adjunctive ultrasound-based technique, has recently been introduced for the assessment of the biomechanical properties of soft tissues. By analyzing tissue deformation in response to applied mechanical stress, strain elastography generates color-coded maps reflecting tissue elasticity and stiffness. Emerging evidence suggests that soft tissue augmentation procedures, particularly those involving connective tissue grafts, may result in increased tissue stiffness over time, which could be indicative of enhanced biomechanical integrity and improved long-term stability of the peri-implant soft tissue phenotype (Table 4) [43].

**Table 4:** Clinical indications of diagnostic tools for evaluating treatment outcomes following soft tissue augmentation AG: attached gingiva; AM: alveolar mucosa; BBT: buccal bone thickness; BBD: buccal bone dehiscence; BOP: bleeding on probing; CAL: clinical attachment level; CBCT: cone beam computed tomography; CRC: complete root coverage; ΔD: linear dimensional change; DH: dentin hypersensitivity; EST: Esthetic Score; GT: gingival thickness; HFUS: high-frequency ultrasonography; IAS: implant aesthetic score; ICAI: Implant Crown Aesthetic Index; IDES: Implant Distal Esthetic score; KM: keratinized mucosa; KT: keratinized tissue; mRC: mean root coverage; MREC: midfacial recession; MT: mucosal thickness; OHIP-14: Oral Health Impact Profile–14; PD: probing depth; PES: Pink Esthetic Score; WES: White Esthetic Score; PROMs: patient-reported outcome measures; PSTD: peri-implant soft tissue dehiscence; RES: Root Esthetic Score; SAT: patient satisfaction; SES: Soft Tissue Esthetic Score; STH: soft tissue height; SUP: suppuration; Vol: volume change; WES: White Esthetic Score Source: [44]

Tool	Primary Indication	Clinical Outcome	Clinical Relevance
Periodontal probe	Routine clinical evaluation	PD, CAL, BOP, KT/KM width, recession depth, PSTD/MREC	First-line tool for assessing soft tissue health, inflammation, and stability
Transmucosal probing	Measurement of soft tissue thickness	GT, MT	Determines phenotype and guides the need for augmentation
CBCT	Hard and soft tissue dimensional analysis	BBT, BBD, STH, peri-implant bone levels	Gold standard for evaluating bone support and 3D tissue architecture
HFUS	Non-invasive soft tissue imaging	GT, MT, BBT, tissue elasticity	Real-time, radiation-free assessment of soft tissue quality and thickness
Superimposition of CBCT + digital impressions	Longitudinal volumetric analysis	Linear changes (ΔD), volumetric changes (Vol), STH	Accurate monitoring of tissue gain/loss over time
Digital impression superimposition	Surface contour evaluation	Profilometric changes, volumetric soft tissue alterations	Ideal for the aesthetic zone and subtle contour changes
Direct clinical assessment	Visual aesthetic evaluation	RES, PES, color match, contour	Immediate chairside evaluation of esthetic outcomes
Photographic analysis	Standardized esthetic documentation	PES/WES, soft tissue harmony	Enables reproducibility and comparison over time
Surveys and questionnaires (PROMs)	Patient-centered evaluation	Pain, morbidity, SAT, OHIP-14	Captures subjective patient experience and quality of life
Doppler ultrasonography / Laser Speckle Doppler	Microvascular assessment	Tissue perfusion and vascularity	Indicates healing potential and graft integration
HFUS (for elasticity)	Tissue biomechanical assessment	Tissue elasticity	Reflects the maturation and functional quality of augmented tissue

Limitations and future perspectives

A limitation of the present review is the absence of a formal systematic review methodology and quantitative evidence synthesis.

Soft tissue augmentation procedures, although highly predictable, are influenced by multiple limitations that can be broadly categorized into patient-related, surgical, and aesthetic factors. Patient-related factors such as smoking, poor oral hygiene, thin gingival phenotype, and systemic conditions (e.g., diabetes) significantly impair vascularity and wound healing, thereby compromising clinical outcomes. Additionally, patient compliance plays a crucial role in maintaining postoperative stability (Table 5).

**Table 5:** Limitations of soft tissue augmentation

Category	Subcategory	Limitation	Clinical Impact
Patient-related factors	–	Smoking	Impaired vascularity, delayed healing, increased risk of graft failure
	–	Poor oral hygiene	Plaque accumulation leading to inflammation and compromised outcomes
	–	Thin gingival phenotype	Reduced tissue stability and higher relapse rates
	–	Systemic conditions (e.g., diabetes)	Delayed wound healing and increased complication risk
	–	Patient compliance	Poor adherence affects healing and long-term stability
Surgical factors	Intraoperative	Improper graft thickness	Affects revascularization and graft survival
		Flap design errors	Inadequate blood supply leading to necrosis
		Excessive graft manipulation/dehydration	Reduced graft vitality
		Tension at the surgical site	Flap necrosis or graft failure
		Inadequate root surface preparation	Poor graft attachment and integration
	Postoperative	Infection or inflammation	Delayed healing and compromised outcomes
		Flap dehiscence/graft exposure	Partial or complete graft loss
		Mechanical trauma (brushing, chewing)	Disruption of the healing graft
		Poor plaque control	Increased inflammation and failure risk
		Inadequate compliance with instructions	Suboptimal healing and outcomes
Aesthetic factors	–	Color mismatch (notably with FGG)	Poor aesthetic integration
	–	Scar formation	Unfavorable tissue appearance
	–	Contour irregularities	Compromised gingival architecture
	–	Incomplete root coverage	Aesthetic dissatisfaction
	–	Graft shrinkage	Reduced long-term esthetic outcomes

Surgical factors may be further divided into intraoperative and postoperative variables. Intraoperative considerations, including graft thickness, flap design, tissue handling, and tension at the surgical site, are critical determinants of graft survival and vascularization. Postoperatively, complications such as infection, flap dehiscence, mechanical trauma, and inadequate plaque control can adversely affect healing and lead to graft failure [45].

From an aesthetic perspective, limitations such as color mismatch (particularly with FGGs), scar formation, contour irregularities, incomplete root coverage, and graft shrinkage may compromise the final clinical outcome despite successful root coverage [46].

Future advances in soft tissue augmentation are likely to be driven by tissue engineering and biomaterials. Next-generation scaffolds combined with growth factors and bioactive molecules may enhance vascularization and integration [43]. Cell-based therapies and smart biomaterials with controlled delivery systems show potential for improving predictability and long-term stability. These innovations may reduce reliance on autogenous grafts by offering minimally invasive, patient-specific alternatives with comparable outcomes. However, further well-designed clinical trials, along with cost-effectiveness and patient-centered evaluations, are needed to support their routine clinical use [44].

## Conclusions

The integration of soft tissue matrices in regenerative periodontal therapy represents a significant advancement in minimally invasive regenerative strategies. While autogenous grafts continue to be the primary modality for many clinicians, STSs offer predictable outcomes with reduced surgical morbidity, making them an ideal option for patients with limited donor tissue or those seeking less invasive interventions. Future research and material innovations will likely continue to refine their properties and broaden their indications, ultimately enhancing clinical outcomes in soft tissue enhancement around both natural dentition and dental implants.

Soft tissue augmentation materials provide effective alternatives to autogenous grafts, reducing patient morbidity while achieving favorable clinical and aesthetic outcomes. Xenogenic collagen matrices and allogenic substitutes demonstrate promising results in increasing soft tissue thickness and keratinized tissue width, though autogenous grafts remain the gold standard. The choice of augmentation material should be guided by defect characteristics, patient factors, and evidence-based outcomes to ensure predictable and stable results.
